# Traditional Chinese Medicine as an adjunct therapy in the treatment of idiopathic membranous nephropathy: A systematic review and meta-analysis

**DOI:** 10.1371/journal.pone.0251131

**Published:** 2021-05-14

**Authors:** Zhenzhen Lu, Wangyi Liu, Hongzhi Gao, Wanjia Chen, Wenshu Ge, Fang Li, Yueyi Deng

**Affiliations:** The Department of Nephrology, Longhua Hospital, Shanghai University of Traditional Chinese Medicine, Shanghai, China; Institute of medical research and medicinal plant studies, CAMEROON

## Abstract

**Background:**

Idiopathic membranous nephropathy (IMN) is one of the most common causes of nephrotic syndrome in adults involving multiple targets and factors. The effect of conservative nonimmunosuppressive or immunosuppressive therapies is unsatisfactory and with many side effects. Traditional Chinese medicine (TCM) can regulate immune function and improve kidney function.

**Purpose:**

To evaluate the total effective rate, curative rate, recurrence rate and adverse events of TCM alone or TCM as an adjunctive therapy for IMN.

**Methods:**

Randomized controlled trials (RCT) comparing either TCM alone or the combination of TCM to western medicine (WM) therapies for patients with IMN were retrieved by searching English and Chinese database. Risk of bias summary was used to assess the methodological quality of eligible studies. Dichotomous data were presented using odds ratios (OR). The primary outcome measure was the total effective rate. Secondary outcomes included curative rate, recurrence rate and adverse events.

**Results:**

29 RCTs involving 1883 participants met the inclusion criteria. There was no statistically significant difference between the therapy of TCM alone and WM on the total effective rates (*OR*: 2.00; 95% *CI*: 0.80–4.98; *P* = 0.14) and curative rate (*OR*: 1.66; 95%*CI*: 0.66–4.22; *p* = 0.28). However, compared to basic treatment or immunosuppressive therapies alone, results showed that TCM as an adjunctive therapy had beneficial effects on the total effective rate (*OR*: 2.59; 95% *CI*: 1.38–4.86; *P* = 0.003 and *OR*: 3.01; 95% *CI*: 2.25–4.04; *P* < 0.00001) and curative rate (*OR*: 3.01; 95%*CI*: 1.24–7.28; *p* = 0.01 and *OR*: 1.73; 95%*CI*: 1.10–2.71; *p* = 0.02). In addition, the combination of TCM treatment could reduce the recurrence rate (*OR*: 0.28; 95% *CI*: 0.12–0.68; *P* = 0.004) and adverse reactions (*OR*: 0.38; 95% *CI*: 0.27–0.54; *p* < 0.00001).

**Conclusion:**

The results indicate that TCM is well-tolerated for the treatment of IMN. However, there remains a need for large-scale and high-quality trials.

## Introduction

Membranous nephropathy (MN) is a common pathological type of adult nephrotic syndrome characterized by the deposition of immune complexes containing antigen, IgG, and complement on the subepithelial side of the glomerular basement membrane (GBM) [1]. Approximate 20%~30% of MN are secondary to systemic causes including infections, malignancy, autoimmune diseases or drugs and the remaining can be identified as idiopathic membranous nephropathy (IMN). It is an autoimmune disease correlated with antibodies against podocyte proteins and about 70% have been reported to be related to M-type phospholipase A2 receptor (PLA2R) [2]. From 2004 to 2014, the proportion of IMN was rose to 23.4% [3] and studies suggested that 30% to 40% of patients progressed toward to end-stage renal failure in 5 to 15 years [4]. According to the different stratification of proteinuria, the available treatment methods for IMN are divided into conservative therapy and corticosteroids and immunosuppressive agents. However, immunosuppressive therapy results in immune tolerance and the existence of multiple complications such as hyperlipidemia, infection, and thromboembolism, making it a major cause of refractory kidney disease. Moreover, a systematic review has showed that immunosuppressive regimen did not improve renal survival and mortality in patients with IMN [5]. Therefore, there exists an urgent need to explore new therapeutic strategies for IMN.

Theory of “yin-yang” and “five elements” as ancient Chinese philosophy were applied to TCM, forming a unique system to diagnose and cure illness. TCM has been proved effective in symptom relief and homeostatic equilibrium in long-term clinical practice [6]. By observing the signs and symptoms, doctors will employ different treatment principles by individual on the basis of syndrome differentiation. A meta-analysis supported that the use of Chinese herbal medicine as a treatment could increase plasma albumin, reduce urine albumin excretion and improve lipid metabolic disorder in the treatment of nephrotic syndrome [7]. For IMN patients, TCM attempts to reduce proteinuria, relieve edema symptoms and prevent complications. Although there are scattered and small clinical trials, a systematic review to provide clinical evidence of TCM in patients with IMN on the efficacy, adverse reactions, and recurrence has not conducted. Thus, it is timely to perform a meta-analysis of TCM for IMN so as to provide evidence for clinical practice.

## Methods

### Data sources and search strategies

Two reviewers (WYL and HZG) systematically searched for RCTs in Chinese database including China National Knowledge Infrastructure (CNKI), Chinese Scientific Journals Database (VIP), Wanfang Data, Sinomed and English database containing pubmed, embase, web of science, Cochrane, clinicaltrial from the construction of the database to 1 February 2020. We used the search strategies containing the comprehensive terms in English databases as following: (medicine, Chinese traditional OR traditional Chinese medicine OR herbal medic* OR Chinese herb* OR plant* OR medicinal plant* OR decoction OR powder OR granules OR complementary medicine or alternative medicine) and (glomerulonephritis, membranous OR membranous nephropathy OR membranous glomerulonephritis OR nephropathy, membranous OR membranous glomerulopathy OR membranous glomerulonephropathy OR idiopathic membranous glomerulonephritis OR idiopathic membranous nephropathy OR heymann nephritis OR nephritis, heymann) and “膜性肾病” or “膜性肾小球肾炎” and“中医药” or “中医” in Chinese databases. There were no limitations of language, document type (journal paper, proceedings and postgraduate theses) or publication status for the trials.

### Inclusion criteria

(1) Types of studies: Only the RCTs were incorporated into the meta-analysis and Quasi-RCTs, non-RCTs were excluded. (2) The Diagnosis of participants: Patients biopsy-proven primary membranous nephropathy (stages 1–4) were included in the study. Secondary membranous nephropathy and rapidly progressive membranous nephropathy were all excluded. (3) Interventions: Studies were available with detailed data and containing at least one of the following comparisons: TCM alone vs. control, TCM with conservative treatment of western medicine vs. conservative treatment of western medicine, or TCM with immunosuppressor vs. immunosuppressor. (4) Outcome Measures: The primary outcome was the total effective rate composed of complete or partial remission which was determined by measuring the level of urinary protein and albumin after treatment. The studies included could have different criteria for judging whether the results were effective. Curative rate defined by the complete remission of IMN, recurrence rate determined by the development of 24-hour proteinuria more than 3.5g after a complete or partial remission, as well as adverse events were all evaluated in the meta-analysis. Study will be excluded for the following reasons: (1) The data of diagnostic criteria, interventions was deficient, particularly the primary outcome cannot be extracted from the clinical trials. (2) The genres of the article were animal experiments, case reports and systematic reviews. (3) There was no control group or control group combined with the treatment of TCM. (4) Full text could not be available.

### Data extraction

Data were extracted by 2 authors (ZZL and WSG) independently based on the inclusive criteria, discrepancies should be further discussed and resolved ultimately by the third author (YYD). Detailed information extracted from the selected studies included the information of first author, year of publication, diagnosis and classification, intervention time, age and gender of participants, the sample size of each group, drop out, the number of complete remission, effective cases, adverse reactions, recurrence and outcome measures.

### Risk of bias assessment

Each of the included RCTs should be strictly evaluated with the Cochrane Collaboration tool for risk of bias, which contains selection, performance, detection, attrition, reporting and other bias. Assessment of studies quality was performed independently by two authors (FL and WJC). Disagreements were resolved by screening and discussing repeatedly and the consistent conclusion was reached by the opinion of a senior author (YYD).

### Data synthesis and analysis

The meta-analysis was carried out using Revman 5.3 software provided by the Cochrane Collaboration [8]. Dichotomous data was expressed as odds ratio (OR) and 95% confidence interval (CI). Statistical heterogeneity was tested by the value of *I*^2^. If the value of *I*^2^ > 50%, randomized-effect model was adopted. Otherwise, the fixed effect model was used. Funnel plot was applied to evaluate the potential reporting bias.

## Results

### Study selection

**Fig 1:**
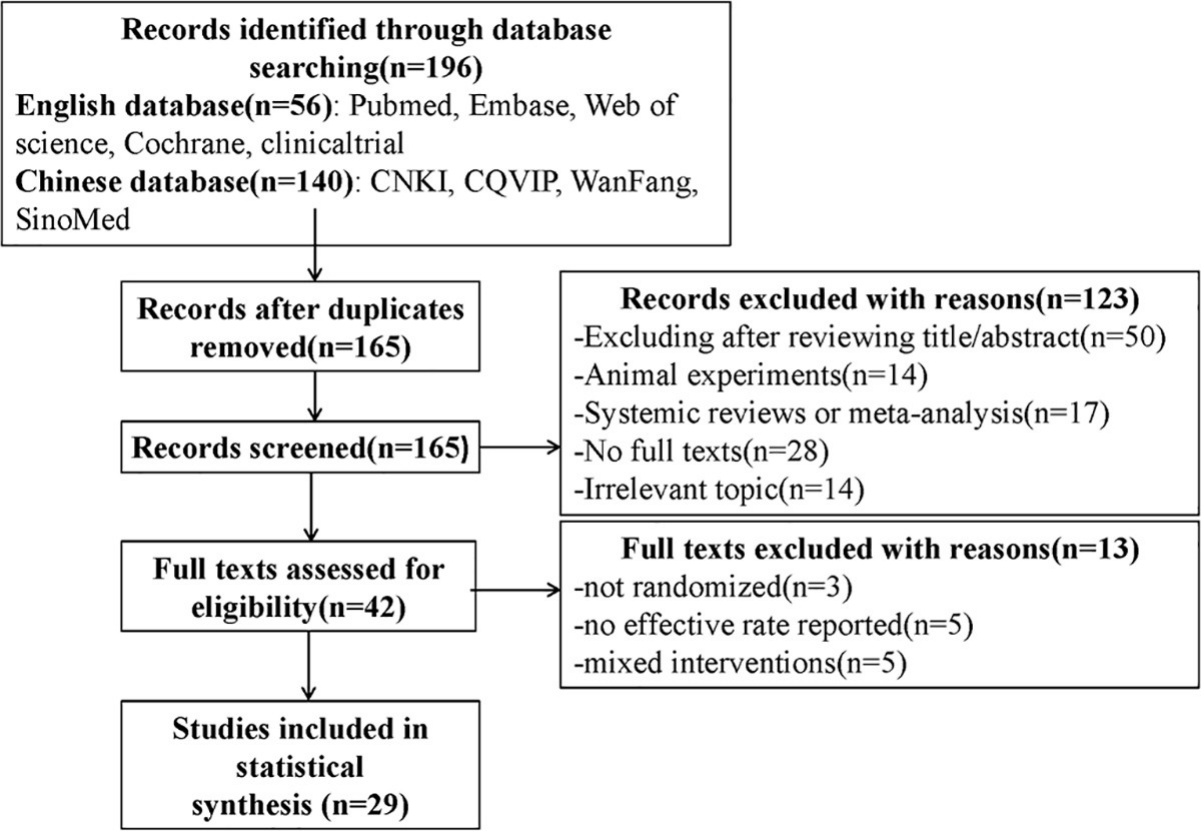
Summary for process of the included studies selection and identification.

An overview of the screening process was summarized in a flow diagram (Fig 1). A total of 196 titles were identified as potentially relevant to the research project after searching nine electronic databases. There remained 165 records after elimination of duplicated articles. Among them, 119 trials were excluded due to the following reasons: (1) after reviewing the title and abstract, (2) animal experiments, (3) systemic reviews or meta-analysis, (4) no full texts, (5) irrelevant topic. Ultimately, the meta-analysis included 29 studies for data synthesis after an overall evaluation of full text. There were 2 trials reported in English and the rest were reported in Chinese.

### Characteristics of eligible studies

29 studies included a sum of 1883 participants, of which 944 participants were in the experimental group and 939 were in the control group respectively [9–37]. The baseline characteristics of participants in different treatment groups were similar in terms of gender and age. With regard of the intervention duration, 3 trials followed up to 12 months [9, 14, 20], 14 trials lasted to 6months [10–13, 16–19, 28–33] 11 trials ranged 2 to 4 months [15, 21–27, 34–36] only 1 trial follow up 1 month [37]. The sample size for both groups is between 15 and 95. TCMs were used in different forms such as pills, powders, decoction, particle and capsule, but they were all administered by oral. Further detailed process of the included studies is presented in Table 1.

**Table 1:** Characteristics of 29 eligible studies.

Study and year	Disease and classification	Intervention time/(months)	Age(year) (M±SD)		Sex (male)		Sample size (n)		Drop out (n)		Interventions		Complete remission (n)		Effective number (n)		Adverse reactions E/C(n)	recurrence		outcome measures
			E	C	E	C	E	C	E	C	E	C	E	C	E	C		E	C	
Chen 2013 [9]	IMN (stages 1–4)	12	49±14	53±12	60	65	95	95	32	26	Shenqi particle	Prednisone+cyclophosphamide	11	20	46	54	YES	NA	NA	①+②+③
Dai 2018 [10]	IMN/NA	6	NA	NA	NA	NA	30	30	NA	NA	QingreHuoxueHushen Decoction +C	prednisone+tacrolimus	NA	NA	22	17	YES	NA	NA	①+③
Diao 2017 [11]	IMN/NA	6	43.96±3.12	43.84 ± 3.08	29	32	50	50	NA	NA	YiqiHuoxueLishui formul+Triptergium wilfordii+C	prednisone	NA	NA	45	34	YES	NA	NA	①+③
Dong 2014 [12]	IMN (stages 1–3)	6	50.83±14.88	51.59±15.33	18	17	30	30	1	2	BushenHuoxue Decoction+C	corticosteroids+cyclophosphamide	7	5	23	22	NA	NA	NA	①+②
Dong 2018 [14]	IMN (stages 1–3)	12	41.96±14.29	44.12±14.75	16	14	30	30	4	4	YiqiHuoxue Decoction+C	tacrolimus	10	6	24	22	YES	5	12	①+②+③+④
Gao 2013 [13]	IMN (stages 1–3)	6	NA	NA	NA	NA	15	15	NA	NA	Tripterygium wilfordii+C	tacrolimus+corticosteroids	4	2	10	7	YES	NA	NA	①+②+③
Guo 2018 [15]	IMN/NA	3	58.7±6.7	58.4±6.5	21	19	36	36	NA	NA	DangguiHuangqi Decoction+C	ARB	NA	NA	34	28	NA	NA	NA	①
Han 2013 [16]	IMN (stages 1–3)	6	NA	NA	NA	NA	35	25	NA	NA	QixueshuiMoshen Decoction	ACEI	4	2	31	22	NA	NA	NA	①+②
Huo 2016 [17]	IMN (stages 1–4)	6	40.50±12.56	41.73±11.73	16	13	23	19	NA	NA	BuqiHuoxueHushen Decoction+C	tacrolimus	6	5	16	12	YES	NA	NA	①+②+③
Jiao 2018 [22]	IMN(stages 1–2)	3	45.43±7.25	46.86±6.79	18	16	30	30	NA	NA	ShenqiZhilong Decoction+C	tacrolimus	7	3	25	19	NA	NA	NA	①+②
Li 2014 [19]	IMN (stages 1–2)	6	28.10±7.26	26.23±6.71	21	20	32	31	NA	NA	JianpiYishenHuoxueQufeng Decoction+C	ACEI	16	4	31	27	NA	NA	NA	①+②
Li 2015 [20]	IMN (stages 1–2)	12	58.4±10.2	58.3±15.0	17	18	30	30	0	1	HuoxueHuayuQushiTongluo Decoction+C	corticosteroids+cyclophosphamide	20	18	28	23	YES	NA	NA	①+②+③
Li 2017 [21]	IMN/NA	4	48.36±9.72	49.39 ± 10.03	18	20	30	30	NA	NA	Shenbing Decoction +C	prednisone	NA	NA	28	21	NA	NA	NA	①
Liu 2018 [18]	IMN (stages 1–4)	6	44.87±10.16	45.21±9.42	23	26	40	40	NA	NA	Shenyankang capsule+HuatanQuyu Decoction	prednisone+cyclophosphamide	27	12	36	26	NA	NA	NA	①+②
Liu and Hu 2019 [23]	IMN/NA	3	45.9±15.0	42.5±15.6	15	20	30	30	NA	NA	Jinguishenqi Pill+Taohongsiwu Decoction +C	ACEI/ARB	NA	NA	25	18	NA	NA	NA	①
Ma 2011 [24]	IMN (stages 1–3)	2	46.07±11.06	48.37±9.70	18	16	30	30	0	0	JiaweiBuyanghuanwu Decoction+C	ACEI/ARB	6	4	24	26	NA	NA	NA	①+②
Ma 2012 [31]	IMN/NA	6	NA	NA	14	12	21	21	NA	NA	HuangqiZhuling Decoction	prednisone	12	8	20	16	YES	NA	NA	①+②+③
Shen 2018 [26]	IMN (stages 1–2)	3	41.00±6.10	42.63±6.31	16	17	30	30	NA	NA	JianpiBushen Decoction+C	prednisone+cyclophosphamide	0	0	28	20	NA	NA	NA	①+②
Sun 2010 [27]	IMN (stages1-4)	2	39.26±7.44	36.50±7.37	12	15	23	26	NA	NA	YiqiYangyinQingliHuoxue formul+C	prednisone+cyclophosphamide	0	0	15	14	YES	NA	NA	①+②+③
Sun 2014 [28]	IMN (stages 2–4	6	40.15±10.05	39.37±11.73	17	19	30	30	NA	NA	Wuzhi capsules+C	tacrolimus+corticosteroids	NA	NA	28	27	NA	NA	NA	①
Wang 2012 [29]	IMN (stages 1–2)	6	NA	NA	NA	NA	45	45	NA	NA	HuoxueQuyu Decoction+C	corticosteroids+ immunosuppressor	NA	NA	43	32	NA	NA	NA	①
Wang 2016 [30]	IMN (stages 1–2)	6	46.67±14.81	48.90±16.16	16	15	30	30	7	5	ShenqiDihuang Decoction+C	tacrolimus+corticosteroids	7	5	17	13	YES	2	8	①+②+③+④
Wang 2018 [34]	IMN/NA	2	43.4±10.5	43.4±8.6	11	10	20	20	NA	NA	GubenXiezhuo particle+C	ARB	7	4	16	10	NA	NA	NA	①+②
Wu 2017 [32]	IMN (stages 1–4)	6	45.47±15.13	47.53±14.46	7	9	15	15	NA	NA	BuqiQufeng Decoction+C	corticosteroids	0	0	13	9	NA	NA	NA	①+②
Xie 2018 [33]	IMN/NA	6	46.1±1.2	45.4±1.5	48	46	90	90	NA	NA	YiqiHuoxueLishui formul+Triptergium wilfordii+C	prednisone+cyclophosphamide	NA	NA	81	61	YES	NA	NA	①+③
Yang 2009 [25]	IMN (stages 1–2)	3	47.17±10.42	48.17±9.14	21	19	36	36	NA	NA	Shenluotong+C	corticosteroids+cyclophosphamide	NA	NA	30	22	NA	NA	NA	①
Yang 2013 [35]	IMN (stages 1–3)	3	53.6±4.3	52.8±3.7	14	15	23	23	NA	NA	GuiqiShengjiang power+ZhengqingfengTongning tablet+C	cyclosporin A	6	4	20	16	NA	2	4	①+②+④
Yu 2014 [37]	IMN/NA	1	48.41±11.30	48.32±11.52	18	19	30	30	NA	NA	YiqiHuoxueLishi Decoction+C	prednisone+cyclophosphamide	11	6	28	24	NA	NA	NA	①+②
Zhang 2017 [36]	IMN (stages 1–3)	2	48±13.64	49.50±9.95	21	18	30	30	1	0	QingxinLianzi Decoction+C	prednisone	2	1	25	20	NA	NA	NA	①+②

E:experimental group; C:control group; NA: no detailed information; ACEI: angiotensin-converting enzyme inhibitor; ARB: angiotensin receptor blocker; ① the total effective rate; ② curative rate; ③adverse events; ④ recurrence rate.

### Risk of bias of included studies

**Fig 2:**
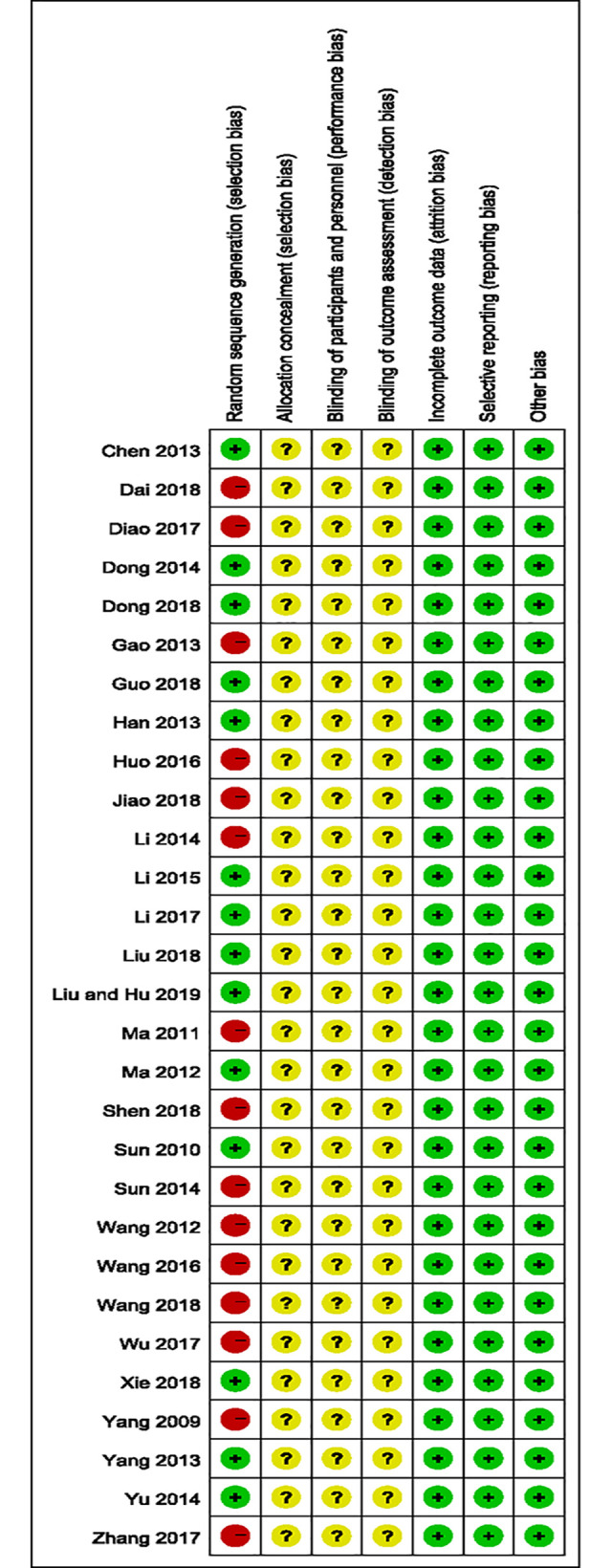
Risk of bias graph: Risk of bias items for each study.

In order to assess the selection, performance, detection, attrition, reporting and other bias of the included studies, Cochrane Collaboration’s risk of bias tool was used and the methodological quality item was shown in Fig 2. The studies included is relatively poor in methodological quality. All of the studies used the principle of randomization and 14 studies described a specific method for random sequence generation including simple random sampling and random number sampling. Only 1 study was reported to use blind methods, but did not specify which method was adopted for blinding of outcome assessment [9]. Therefore, potential performance bias and detection biases could not be ruled out. All the studies were parallel and controlled clinical trials and reported follow-up outcome data, in which 7 studies mentioned the number of withdrawal and drop-out [9, 12, 14, 20, 24, 30, 36]. Selective reporting was fully addressed in all studies. We find no other bias of all studies through the information provided.

### Primary outcomes

#### The total effective rate of TCM alone versus western medicine

**Fig 3:**
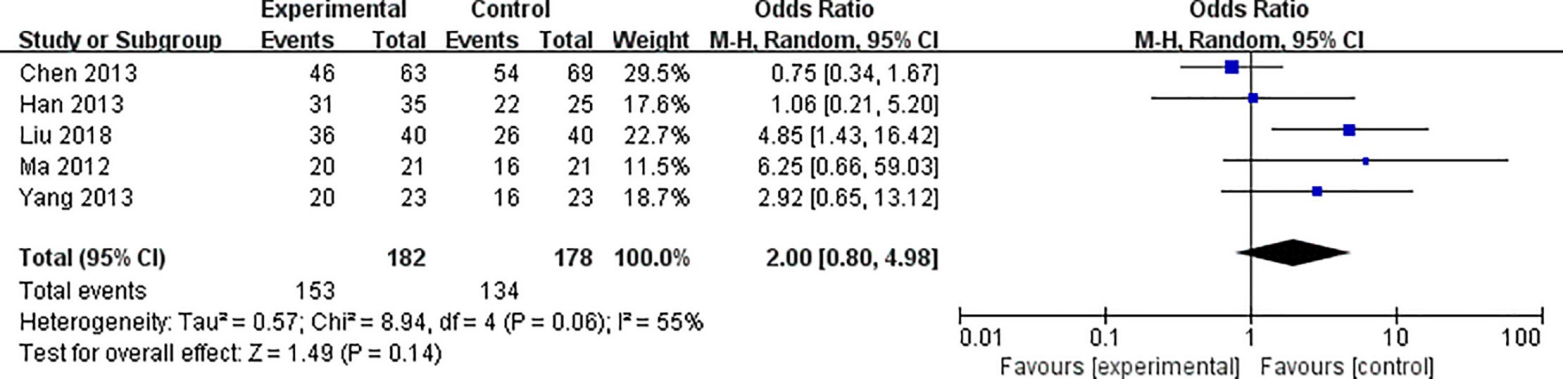
The total effective rate of TCM alone versus western medicine: Forest plot of comparison of the included trials.

5 trials containing 360 participants compared TCM alone to western medicine treatment in terms of the total effective rate [9, 16, 18, 31, 35]. We use a random effect model due to the greater heterogeneity (*χ*^2^ = 8.94, *P* = 0.06, *I*^*2*^ = 55%). The results showed that there was no significant difference between TCM alone and western medicine treatment (*OR*: 2.00; 95% *CI*: 0.80–4.98; *P* = 0.14) (Fig 3).

#### The total effective rate of TCM plus basic treatment versus basic treatment

**Fig 4:**
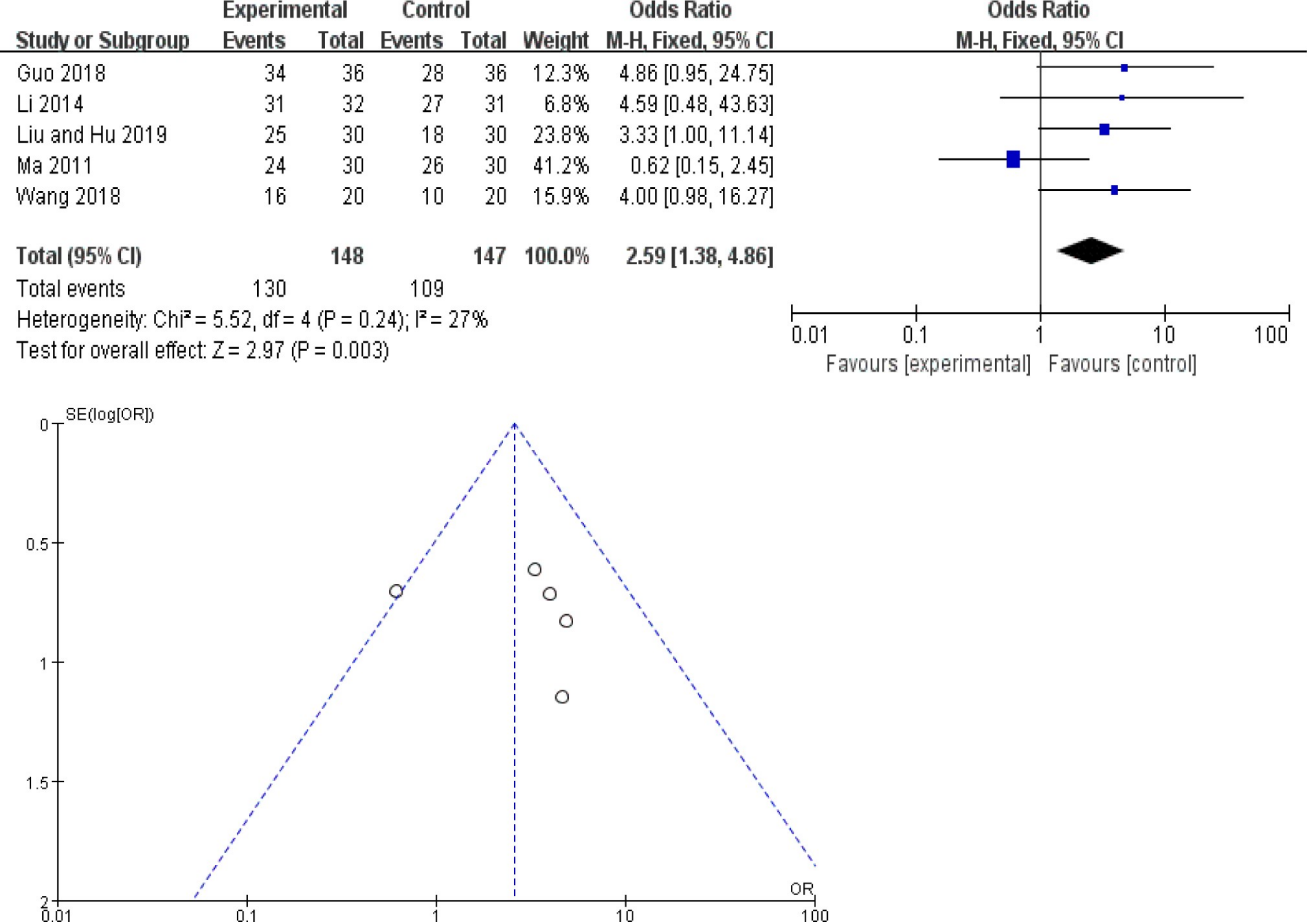
The total effective rate of TCM plus basic treatment versus basic treatment: (A) forest plot; (B) funnel plot.

There were 5 RCTs including 295 cases in the analysis [15, 19, 23, 24, 34]. Based on the same basic treatment of control group, experimental group combined the therapy of TCM and compared the clinical total effective rate between the two groups. The heterogeneity was shown in the analysis (*χ*^2^ = 5.52, *P* = 0.24, *I*^2^ = 27%). The result showed a significantly better efficacy of TCM plus basic treatment in the total effective rate compared to basic treatment alone (*OR*: 2.59; 95% *CI*: 1.38–4.86; *P* = 0.003), the funnel plot was roughly symmetric (Fig 4).

#### The total effective rate of TCM plus immunosuppressor versus immunosuppressor

**Fig 5:**
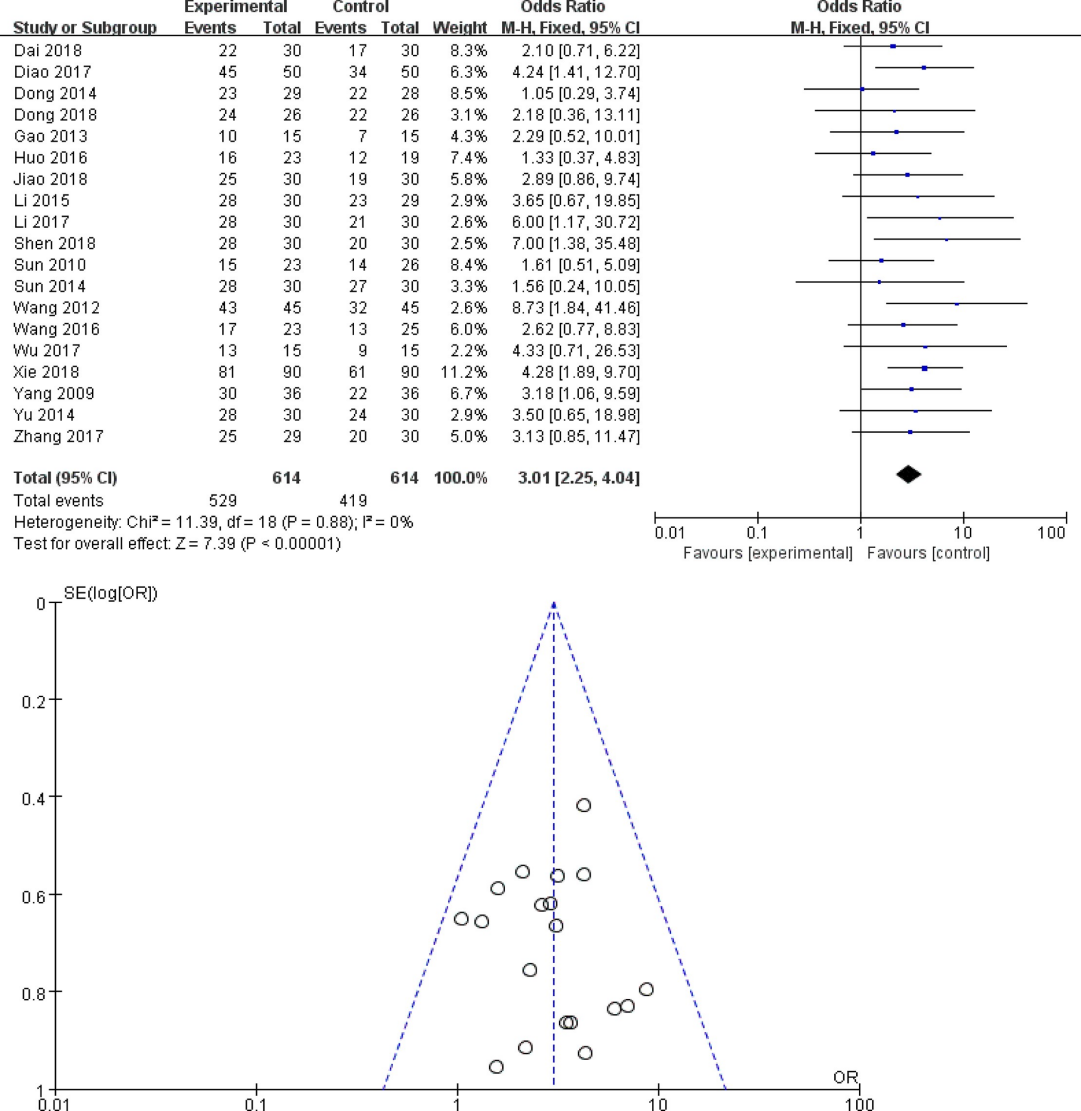
The total effective rate of TCM plus immunosuppressor versus immunosuppressor: (A) forest plot; (B) funnel plot.

19 trials compared the effect of TCM combined with immunosuppressive therapy to immunosuppressive therapy alone [10–14, 17, 20–22, 25–30, 32, 33, 36, 37]. A fixed effect model was applied since trials showed heterogeneity in the consistency (*χ*^2^ = 11.39, *P* = 0.88; *I*^2^ = 0%). Compared to immunosuppressive therapy alone, TCM adjuvant therapy significantly improved the total effective rate(*OR*: 3.01; 95% *CI*: 2.25–4.04; *P* < 0.00001) (Fig 5). It was suggested that TCM as a complementary therapy was beneficial to improve clinical effectiveness in patients with IMN.

### Secondary outcomes

#### Curative rate

**Fig 6:**
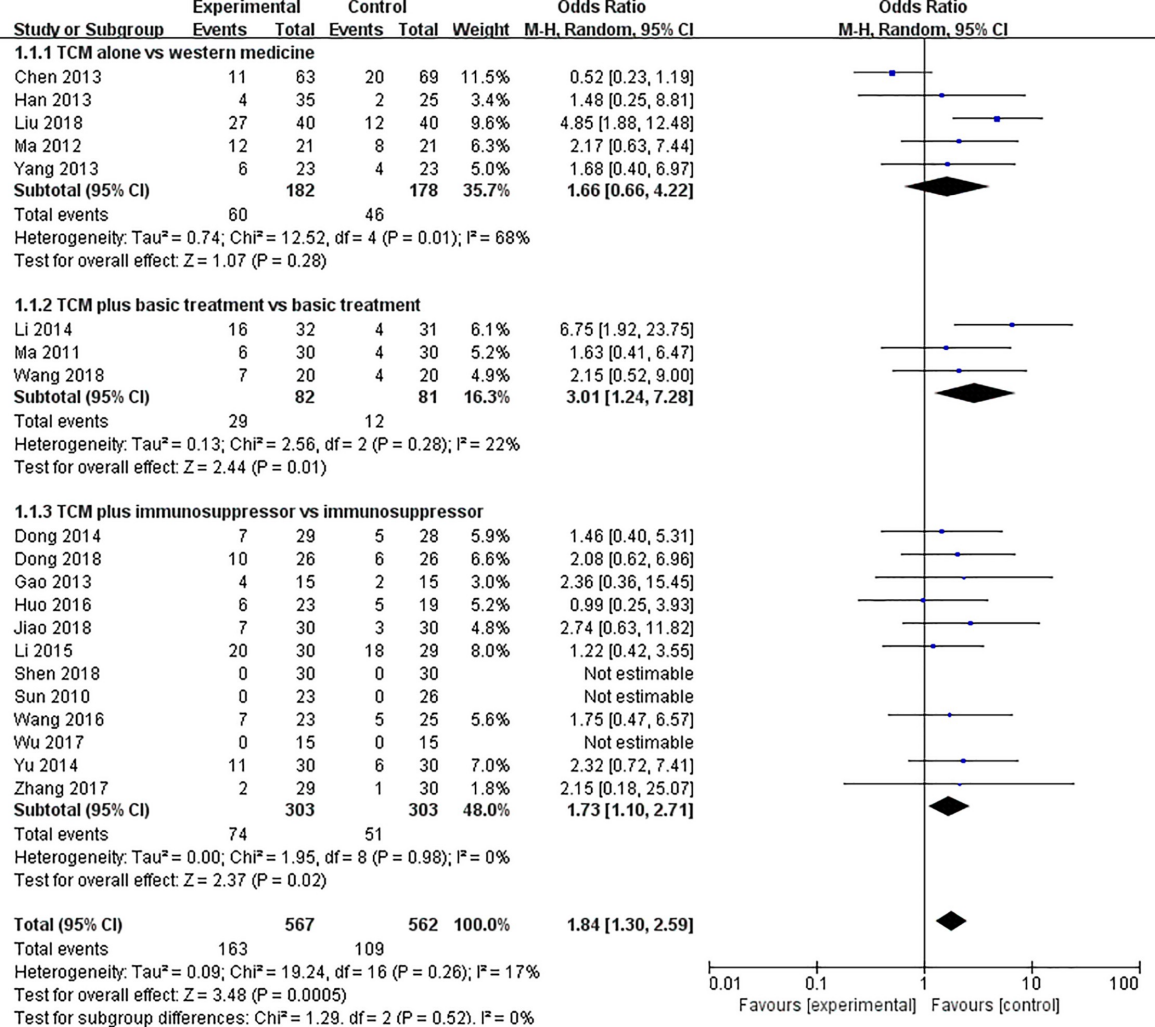
The curative rate of TCM alone or TCM combination of western medicine versus western medicine alone.

In this outcome analysis, we performed a subgroup analysis containing 20 studies [9, 12–14, 16–20, 22, 24, 26, 27, 30–32, 34–37] (*OR*: 1.84; 95% *CI*: 1.30–2.59; *p* = 0.0005), which were divided into 3 categories according to different therapy regimen. The corresponding results of each group are as following: TCM alone vs. western medicine treatment (*OR*: 1.66; 95%*CI*: 0.66–4.22; *p* = 0.28), TCM plus basic treatment vs. basic treatment (*OR*: 3.01; 95%*CI*: 1.24–7.28; *p* = 0.01), TCM plus immunosuppressive agents vs. immunosuppressive agents alone (*OR*: 1.73; 95%*CI*: 1.10–2.71; *p* = 0.02) (Fig 6). Only the first of the studies showed no statistical significance between the two groups. The remaining two trials reported that there was a significant difference between the integration of TCM and western medicine compared with western medicine treatment alone on the curative rate.

#### Recurrence rate

**Fig 7:**
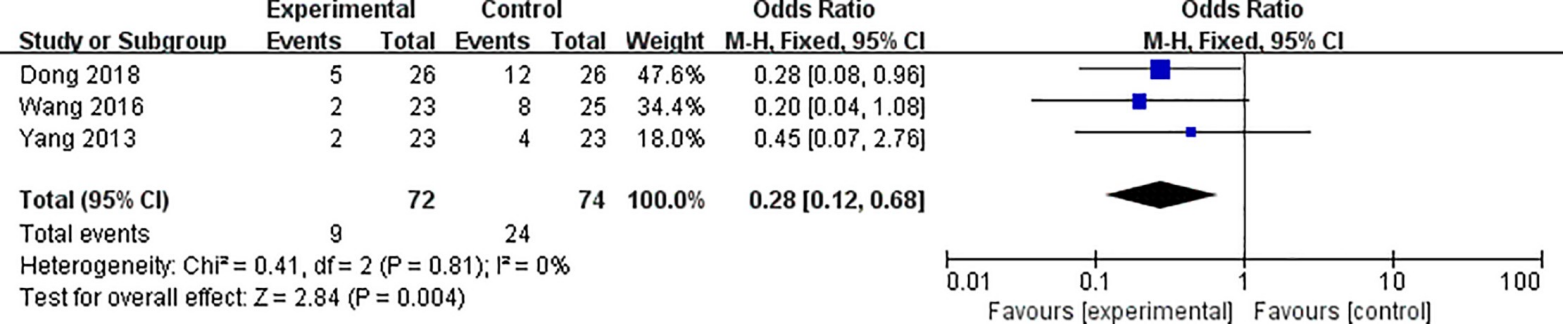
The recurrence rate of TCM alone or TCM combination of western medicine versus western medicine alone.

Only 3 trials described the number of relapses during follow-up in the experimental and control groups [14, 30, 35]. The results showed that the recurrence rate of TCM alone or TCM combined with western medicine treatment was significantly lower than western medicine treatment alone (*OR*: 0.28; 95% *CI*: 0.12–0.68; *P* = 0.004) (Fig 7).

#### Adverse events

**Fig 8:**
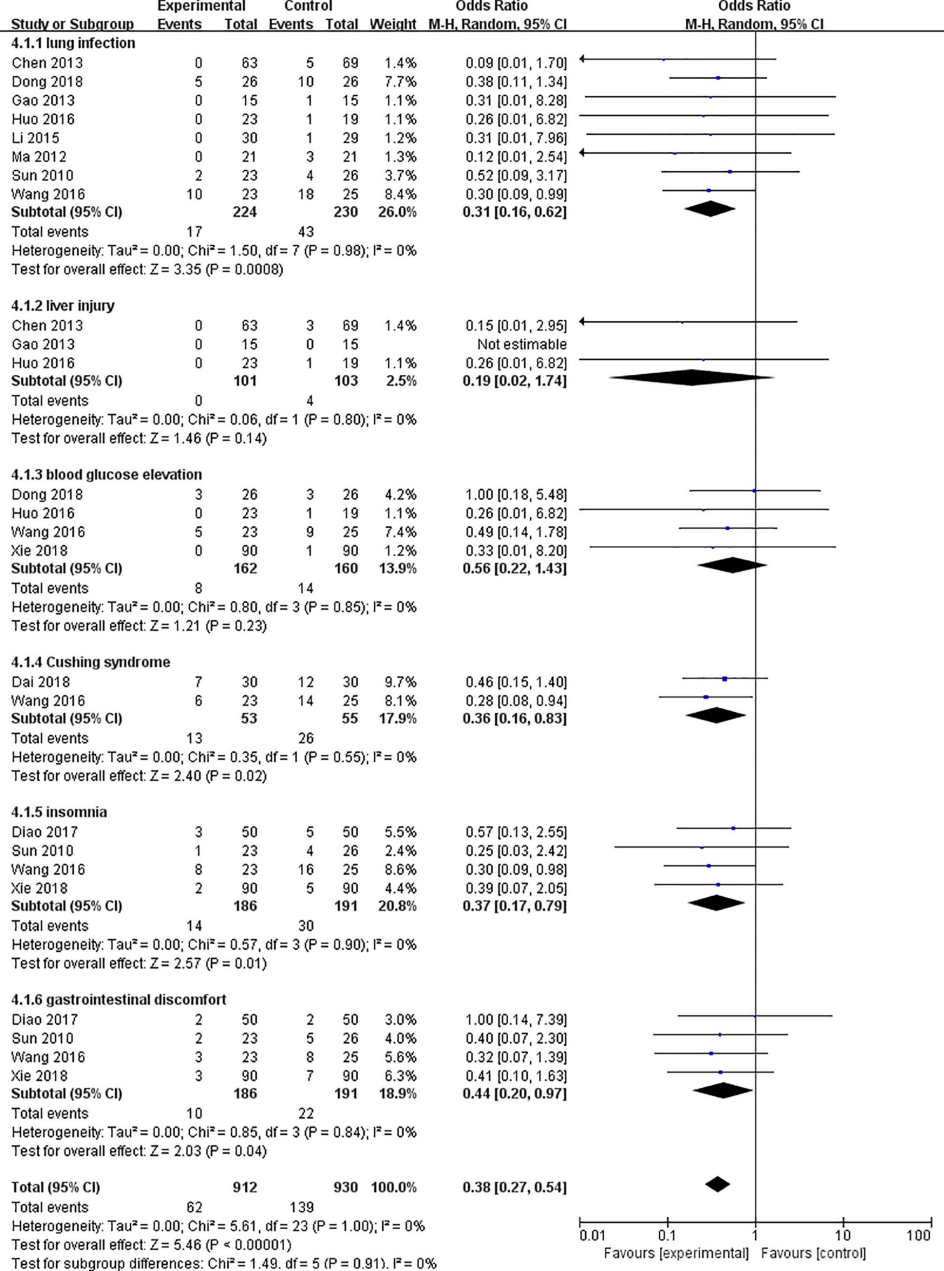
The adverse events of TCM alone or TCM combination of western medicine versus western medicine alone.

11 trials mentioned adverse events during the follow-up [9–11, 13, 14, 17, 20, 27, 30, 31, 33]. The side effects could be summarized as following: lung infection (*OR*: 0.31; 95% *CI*: 0.16–0.62; *P* = 0.0008), liver injury (*OR*: 0.19; 95% *CI*: 0.02–1.74; *P* = 0.14), blood glucose elevation (*OR*: 0.56; 95%*CI*: 0.22–1.43; P = 0.23), Cushing syndrome(*OR*: 0.36; 95%*CI*: 0.16–0.83; *P* = 0.02), insomnia (*OR*: 0.37; 95%*CI*: 0.17–0.79; *P* = 0.01), gastrointestinal discomfort (*OR*: 0.44; 95% *CI*: 0.20–0.97; *P* = 0.04) (Fig 8). The result indicated that TCM may have a good effect on reducing lung infection, Cushing syndrome, insomnia and gastrointestinal discomfort events significantly. However, compared with the western medicine control group, TCM did not seem to decrease the occurrence of liver injury and blood glucose elevation events (Fig 8).

## Discussion

Several system reviews have been conducted to evaluate the efficacy of TCM therapy from clinical indicators such as creatinine, urinary protein and albumin. To our knowledge, the present meta-analysis is the first attempt to evaluate the safety and efficacy of TCM treatment from a more comprehensive perspective of the total effective rate, curative rate, recurrence rate and adverse events.

### Summary of main results

29 trials were included in the analysis to evaluate the effects of TCM on IMN. The results provide evidence that TCM as an adjunct therapy has favorable therapeutic benefits and serves to mitigate some adverse reactions in some cases. Compared with basic treatment or immunosuppressive therapy alone, the integration of TCM and western medicine regimen for the patients of IMN can improve the total effective rate and curative rate. The use of TCM alone showed no statistical significance compared with western medicine treatment on the total effective rate and curative rate. We supposed that insufficient sample size and mixed intervention methods are remained the major reasons. Although only 3 studies described the number of recurrence in the process of trials, the result demonstrated that the rate of recurrence in TCM combination group was lower than that of western medicine group. Furthermore, we extracted the data of adverse events in 11 trials including lung infection, liver injury, blood glucose elevation, Cushing syndrome, insomnia, gastrointestinal discomfort. It is shown that TCM may reduce the incidence of infection by regulating the body’s immunity and has more advantages in alleviating symptoms of insomnia, gastrointestinal discomfort and Cushing’s syndrome. However, we did find TCM combined group was better than control group in improving the liver injury and blood glucose elevation. Remarkably, we observed transient liver enzyme elevation in the TCM combined group in some studies, but the liver function returned to normal after corresponding treatment.

### Possibility and rationality of TCM for the treatment of IMN

Although it is generally accepted that IMN is a renal-limited autoimmune disease, which is identified in 70–80% of patients with antibodies against PLA2R (aPLA2Rab), many detailed questions associated with the disease such as genetic susceptibility, environmental factors, the relevant B- and T-cell epitopes and the action mechanisms of podocyte injury remain unsolved [38]. According to the “Improving Global Outcomes guidelines [39]”, patients should be eligible for immunosuppressive therapies if the estimated glomerular filtration rate decreases or the level of proteinuria or aPLA2Rab is severe after 6 months conservative therapy. However, immunosuppressive agents exert a number of dose-dependent and age-dependent side effects such as infection, diabetes mellitus, osteoporosis hypertension, obesity-metabolic syndrome, peptic ulcer in the elderly [40], in which IMN often occurs. Moreover, these agents are related to a high incidence of relapse after discontinuation [41]. Thus, measures should be taken to prevent or attenuate these untoward events and relapse. TCM is composed of different components according to the principle of “Jun Chen Zuo Shi,” which can exert a synergistic effect as a whole rather than a single molecular target. By observing the clinical manifestations of IMN, it is mostly attributed to "edema" and the main pathogenesis explained by TCM is deficiency in root and excess in superficiality. Deficiency in root is mainly attributed to deficiency of spleen and kidney. Traditional Chinese medicine believed that spleen deficiency could not carry out the function to transport and transfer water dampness, causing water dampness to stay in the body, overflowing skin to form edema. Kidney deficiency led to impaired function of kidney storage and leakage of substance essence, proteinuria occuring in the process of disease. The accumulation of water dampness and metabolic waste to become turbid toxicity in the body. Water dampness blocked the movement of Qi, Qi stagnation could not move blood and formed blood stasis. Therefore, the excess in superficiality is mainly water dampness, turbid toxicity and blood stasis. As a result, the treatment principles are summarized to strengthen the spleen, tonify the kidney to cure the root and remove the dampness and toxicity to cure the superficiality. In addition, activating blood and dissolving stasis should be adopted through the whole progression of the treatment of IMN. Shenqi Particle based on the traditional Chinese medicine theory of balance “yin-yang” and reduce “dampness” and excessive “heat” was proven effective and had fewer side effects compared to standard therapy of prednisone and cyclophosphamide in a multicenter randomized controlled clinical trial, which obtained high clinical recognition in the field of TCM treatment of MN [9]. The course of IMN includes spontaneous remission, acute progression and slow development. Modern medicine has no specific markers to predict different outcome of the diseases. In order to provide the evidence about the effectiveness of TCM as an alternative therapy for IMN, the systematic review and meta-analysis was performed. Our results suggested that TCM can help to improve effective rate and mitigate adverse events of the application of western medicine. We can make a routine recommendation of TCM for IMN treatment in accordance with the results as followed. For the patients with a tendency of spontaneous remission or slow development, the combination of TCM with basic treatment can promote the body’s immune response to a balanced state based on the holistic concept, which plays an important role in maintenance of internal environment. Patients of IMN presented as acute progression, TCM integrated to immunosuppressive therapy not only can enhance the therapeutic efficacy, but also can mitigate or reduce side effects and recurrence cause by corticosteroids and immunosuppressor.

### Limitations

Though this study supports the positive role of TCM as an alternative treatment for IMN, there are still some limitations to this meta-analysis. First of all, the major shortcoming of our study is the methodology of the quality of 29 studies included was generally poor due to small sample size, lack of long term follow-up and meticulous design. While all of the trials demonstrated randomization, only 14 trials described concrete methods for random sequence generation. 1 trial referred to adopting blind methods, but did not specify what kind of blindness was used. These questions mentioned above increased the selection bias and caused performance bias and detection bias unclear. Furthermore, all participants incorporated in the study were Chinese. We should validate the efficacy of TCM in treating IMN in other countries to eliminate the influence of race in further study. The last but not the least, the results revealed that TCMs integrated western medicine may play a beneficial role on the treatment of IMN. However, detailed administration information of prescriptions on dosage, form, indications were all absent and most of the studies included had not conducted in-depth research on the action mechanism of the role of TCM. Therefore, considering these limitations, exquisite design and high quality clinical practice must be done cautiously to provide more evidence in the wide application of TCM for IMN.

## Conclusions

In spite of the limitations of the small sample sizes, low quality and imperfect design of the included studies, the meta-analysis provided reliable evidence on the potential advantages including higher total effective rate, curative rate and fewer recurrence rate, adverse reactions of TCM as an alternative treatment for IMN, especially in the absence of effective therapies of modern medicine. Therefore, we recommend that TCM treatment of MN is a promising alternative therapy. However, TCM doctors mostly used empirical prescriptions clinically, and there is a lack of standardization of TCM treatment prescriptions. In the future, the refined designed RCTs are required for assessing the effect of prescriptions for IMN and experimental studies need to clarify the mechanism of effectiveness.

## Supporting information

S1 Checklist PRISMA checklist. (DOC)

S1 File Cover letter. (PDF)

## Abbreviations

IMN: Idiopathic Membranous Nephropathy
MN: Membranous Nephropathy
TCM: Traditional Chinese Medicine
RCT: Randomized Controlled Trials
WM: Western Medicine
OR: Odds Ratios
PLA2R: M-type Phospholipase A2 Receptor
CNKI: China National Knowledge Infrastructure
VIP: Chinese Scientific Journals Database
CI: confidence interval
E: experimental group
C: control group
NA: no detailed information
ACEI: angiotensin-converting enzyme inhibitor
ARB: angiotensin receptor blocker

## References

[1] Yu Zhang, Yabin Jin, Zhanwen Guan et al. The Landscape and Prognosis Potential of the T-Cell Repertoire in Membranous Nephropathy.[J]. Front Immunol, 2020, 11: 387. 10.3389/fimmu.2020.00387 32210970 PMC7076165

[2] Sonia Boyer-Suavet, Marine Andreani, Maël Lateb et al. Neutralizing Anti-Rituximab Antibodies and Relapse in Membranous Nephropathy Treated With Rituximab.[J].Front Immunol, 2019, 10: 3069. 10.3389/fimmu.2019.03069 31998325 PMC6970431

[3] Xin Xu, Guobao Wang, Nan Chen et al. Long-Term Exposure to Air Pollution and Increased Risk of Membranous Nephropathy in China.[J].J. Am. Soc. Nephrol., 2016, 27: 3739–3746. 10.1681/ASN.2016010093 27365535 PMC5118492

[4] GLASSOCK R. Diagnosis and natural course of membranous nephropathy[J]. Seminars in Nephrology, 2003, 23(4):324–332. 10.1016/s0270-9295(03)00049-4 12923720

[5] Perna A, Schieppati A, Zamora J, et al. Immunosuppressive treatment for idiopathic membranous nephropathy: A systematic review[J]. american journal of kidney diseases the official journal of the national kidney foundation, 2004, 44(3):385–401. 15332211

[6] Zhenzhen Lu, Yifei Zhong, Wangyi Liu et al. The Efficacy and Mechanism of Chinese Herbal Medicine on Diabetic Kidney Disease.[J].J Diabetes Res, 2019, 2019: 2697672. 10.1155/2019/2697672 31534972 PMC6732610

[7] Feng M, Yuan W, Zhang R, et al. Chinese herbal medicine Huangqi type formulations for nephrotic syndrome[J]. Cochrane Database of Systematic Reviews, 2013, 6(6):CD006335. 10.1002/14651858.CD006335.pub3 23740567 PMC11380091

[8] Nordic Cochrane Centre, Review Manger (RevMan) [Computer Program]. Version 5.3, The Nordic Cochrane Centre, The Cochrane Collaboration, Copenhagen, Denmark, 2014.

[9] Chen Y, Deng Y, Ni Z, et al. Efficacy and Safety of Traditional Chinese Medicine (Shenqi Particle) for Patients With Idiopathic Membranous Nephropathy: A Multicenter Randomized Controlled Clinical Trial[J]. American Journal of Kidney Diseases, 2013, 62(6):1068–1076. 10.1053/j.ajkd.2013.05.005 23810688

[10] Dai M. and Zhu Y., “Discussion on the effect of Qingre Huoxue Yishen decoction in the adjuvant treatment of membranous nephropathy”, Contemporary Medical Symposium, 2018, 016(024):186–188.

[11] Diao X. M.,” Effect of Yiqi HuoxueLishui combined with tripterygium wilfordii polyglycosides on membrane nephropathy and PCX expression”, Modern Journal of Integrated Traditional Chinese and Western Medicine, 2017(25):90–92.

[12] Dong L., “Clinical study on the treatment of primary membranous nephropathy with tonifying kidney and activating blood circulation based on the theory of collateralization”, Shandong Traditional Chinese Medicine University, 2014.

[13] Gao F., “Efficacy of low dose hormone combined with tacrolimus and tripterygium wilfordii polyglycoside tablets in the treatment of idiopathic membranous nephropathy”, International Journal of Transplantation and Hemopurification, 2013, 011(002):30–33.

[14] Dong L. P., “Clinical observation on treatment of PMN with tacrolimus and Yiqi Huoxue prescription”, Henan university of traditional Chinese medicine, 2018.

[15] Guo S. L. and Jiang S., “Analysis of the application value of angelica astragalus decoction combined with omesartan in patients with low risk of idiopathic membranous nephropathy”, Clinical Journal of Chinese Medicine,2018,10(08):125–126.

[16] Han D. Y. and Zhao C., “Clinical observation of treating idiopathic membranous nephropathy with qi, blood and water”, Chinese Journal of Basic Medicine in Traditional Chinese Medicine, 2013, 019(011):1311–1313.

[17] Huo S. and Feng R., “Clinical value of Buqi Huoxue Hushen prescription combined with tacrolimus in the treatment of idiopathic membranous nephropathy”, Chinese Journal of Integrated Traditional and Western Nephrology, 2016, 17(8):714–716.

[18] Liu X. Y. and Che Y. J., “Evaluation of therapeutic effect of huayan quyu decoction on idiopathic membranous nephropathy with Yang deficiency of spleen and kidney”, Nei Mongol Journal of Traditional Chinese Medicine,2018,37(12):28–29.

[19] Li X. and Lu B.,” Clinical study on the treatment of idiopathic membranous nephropathy by invigorating spleen, invigorating kidney, activating blood and dispelling wind”, Journal of New Chinese Medicine,2014,46(05):73–75.

[20] Li Q. F.,” The academic experience of cheng xiaoxia and her application in the treatment of membranous nephropathy”, Zhejiang Chinese Medical University, 2016.

[21] Li Y. J.,” Clinical observation of nephrotic prescription combined with western medicine in the treatment of primary nephrotic syndrome with spleen-kidney deficiency”, Shenzhen journal of integrated traditional Chinese and western medicine,2017,27(23):25–27.

[22] Jiao Z. S., “Effect observation of shenqi Zhilong decoction in the treatment of membranous nephropathy with qi deficiency and blood stasis”, Heilongjiang academy of Chinese medicine, 2018

[23] Liu C. Y. and Hu J. F.,” The effect of warming kidney and dredging collaterals on the clinical effect and urinary C5b-9 in patients with idiopathic membranous nephropathy”, International Journal of Traditional Chinese Medicine, 2019, 41(3):229–233.

[24] Z. W. M, “Observation on idiopathic membranous nephropathy of blood stasis due to qi deficiency with jia-wei-bu-yang-huan-wu powder”, Shandong Traditional Chinese Medicine University,2011.

[25] Yang F. W., “Clinic observation of treating primariy membranous nephropathy patients with shenluotong and effect to the function of endotheliocyte”, hebei medical university,2009.

[26] Shen X. N., “Clinical Observation on Treatment of Stage I and II Idiopathic Membranous Nephropathy (Spleen Kidney Yang Deficiency Type) with Jianpi Bushen Prescription”, Henan university of traditional Chinese medicine,2018.

[27] Sun Y.H.,” By Yiqi Yangyin and Qingli Huoxue–based treatment of recurrent membranous nephropathy (qi and Yin deficiency and heat stasis card) clinical observation”, Chengdu University of TCM,2010.

[28] Sun Z, Ren M, Wu Q, et al. Co-administration of Wuzhi capsules and tacrolimus in patients with idiopathic membranous nephropathy: clinical efficacy and pharmacoeconomics[J]. International Urology & Nephrology, 2014, 46(10):1977–1982. 25145781 10.1007/s11255-014-0801-3

[29] Wang H. J. and Zhang S. M. et al., “Live blood stasis and eliminating turbidity treatment of membranous nephropathy”, China Journal of Pharmaceutical Economics,2012(06):115–117.

[30] Wang Q. L.,” Clinical study of shenqidhuang decoction combined with tacrolimus and low dose hormone in the treatment of idiopathic membranous nephropathy”, Shandong Traditional Chinese Medicine University,2016.

[31] Ma C. W. and Xie M. H., “Huangqi Zhuling decoction was used to treat 21 cases of refractory membranous nephropathy”, Traditional Chinese Medicinal Research, 2012,25(11):38–40.

[32] Wu Q. F., “clinical observation and theoretical exploration of Buqi Qufeng method in the treatment of membranous nephropathy”, Nanjing University of Traditional Chinese Medicine,2017.

[33] Xie X. and Zhan J. H. et al.,” Study on the effect of tripterygium wilfordii polyglycosides, invigorating qi and promoting blood circulation combined with western medicine on the treatment of idiopathic membranous nephropathy and the expression of PCX”, Chinese Journal of Control of Endemic Diseases,2018,33(01):63–64.

[34] Wang Y. H. and Shen B. L. et al., “Clinical observation of Guben Xiezhuo Granule in Treatment of Idiopathic Membranous Nephropathy with Qi Deficiency and Dampness Obstruction Syndrome”, Acta Chinese Medicine,2018,33(05):882–886.

[35] Gao J. J. and Yang H. J. et al.,” Observation on the curative effect of Guiqi Shengsan and Zhengqing Fengtongning sustained release tablets in the treatment of refractory membranous nephropathy”, Hebei Journal of Traditional Chinese Medicine,2013,35(08):1168–1170.

[36] Xiang Z. L. and Chen D. H., “Clinical study of qingxinlianzi decoction in the treatment of deficiency of qi and Yin in membranous nephropathy of primary nephrotic syndrome in hormone withdrawal stage”, Asia-Pacific Traditional Medicine, 2017,13(15):151–153.

[37] Yu L. Q. and Feng C. J. et al.,” Clinical observation on the treatment of membranous nephropathy (dampness-heat syndrome) with traditional Chinese medicine of invigorating qi, activating blood and induing moistening”, Journal of Emergency in Traditional Chinese Medicine,2014,23(07):1324–1325.

[38] van de Logt Anne-Els, Fresquet Maryline, Wetzels Jack F et al. The anti-PLA2R antibody in membranous nephropathy: what we know and what remains a decade after its discovery. [J].Kidney Int., 2019, 96: 1292–1302. 10.1016/j.kint.2019.07.014 31611068

[39] Management and treatment of glomerular diseases (part 1): conclusions from a Kidney Disease: Improving Global Outcomes (KDIGO) Controversies Conference[J]. Kidney International, 2019, 95(2):268–280. 10.1016/j.kint.2018.10.018 30665568

[40] Claudio Ponticelli, Rachele Escoli, Gabriella Moroni, Does cyclophosphamide still play a role in glomerular diseases?[J].Autoimmun Rev, 2018, 17: 1022–1027. 10.1016/j.autrev.2018.04.007 30107267

[41] Fervenza Fernando C, Appel Gerald B, Barbour Sean J et al. Rituximab or Cyclosporine in the Treatment of Membranous Nephropathy.[J].N. Engl. J. Med., 2019, 381: 36–46. 10.1056/NEJMoa1814427 31269364

