# Supplementary material for: Traditional Chinese Medicine as an adjunct therapy in the treatment of idiopathic membranous nephropathy: A systematic review and meta-analysis
Source: PLoS One. 2021 May 14;16(5):e0251131. doi: 10.1371/journal.pone.0251131 (PMC8121368; doi:10.1371/journal.pone.0251131)
Supplement: S1 Checklist — (DOC) [file pone.0251131.s001.doc]

| **Section/topic** | **#** | **Checklist item** | **Reported on page #** |
| --- | --- | --- | --- |
| **TITLE** | | | page1 |
| Title | 1 | The report is identified as a systematic review and meta-analysis. | title |
| **ABSTRACT** | | | page1-2 |
| Structured summary | 2 | The structured abstract includes background; objectives; methods; results; conclusions. | Abstract, paragraph 1-4 |
| **INTRODUCTION** | | | page2-3 |
| Rationale | 3 | Described the rationale for the review in the context of what is already known. | Introduction, paragraph 1 |
| Objectives | 4 | Provided an explicit statement of questions in the introduction. | Introduction, paragraph 2 |
| **METHODS** | | | Page3-5 |
| Protocol and registration | 5 | The protocol is described in the Method. The registration number does not apply. | Method, paragraph 1-5 |
| Eligibility criteria | 6 | *The part of Inclusion Criteria* specified study characteristics and report characteristics used as criteria for eligibility. | Method, paragraph 2 |
| Information sources | 7 | *The part of Data Sources and Search Strategies* described all information sources in the search and date last searched. | Method, paragraph 1 |
| Search | 8 | Present full electronic search strategy, so that it could be repeated. | Method, paragraph 1 |
| Study selection | 9 | State the process for selecting studies. | Result, paragraph 1 |
| Data collection process | 10 | Describe method of data extraction from reports and any processes for obtaining and confirming data from investigators. | Method, paragraph 3 |
| Data items | 11 | List and define all variables for which data were sought and any assumptions and simplifications made. | Method, paragraph 2 |
| Risk of bias in individual studies | 12 | Describe methods used for assessing risk of bias of individual studies. | Result, paragraph 3 |
| Summary measures | 13 | State the principal summary measures. | Method, paragraph 5 |
| Synthesis of results | 14 | Describe the methods of handling data including measures of consistency for each meta-analysis. | Method, paragraph 5 |

Page 1 of 2

| **Section/topic** | **#** | **Checklist item** | **Reported on page #** |
| --- | --- | --- | --- |
| Risk of bias across studies | 15 | Specify any assessment of risk of bias that may affect the cumulative evidence. | Result, paragraph3 |
| Additional analyses | 16 | Describe methods of subgroup analyses. | Result, paragraph 5 |
| **RESULTS** | | | Page5-7 |
| Study selection | 17 | Give numbers of studies screened, assessed for eligibility, and included in the review, with reasons for exclusions at each stage, ideally with a flow diagram. | Result, paragraph1 |
| Study characteristics | 18 | For each study, the data of study size, the time of follow-up were extracted and provide the citations. | Result, paragraph2 |
| Risk of bias within studies | 19 | Present data on risk of bias of each study and level assessment. | Result, paragraph3 |
| Results of individual studies | 20 | For all outcomes considered presented simple summary data for each intervention group, effect estimates and confidence intervals, ideally with a forest plot. | Result, paragraph1-5 |
| Synthesis of results | 21 | The results of each analysis including confidence intervals and measures of consistency were presented. | Result, paragraph1-5 |
| Risk of bias across studies | 22 | Presented results of assessment of risk of bias across studies. | Result, paragraph3 |
| Additional analysis | 23 | Additional analyses of subgroup analyses were done. | Result, paragraph 5 |
| **DISCUSSION** | | | Page7-9 |
| Summary of evidence | 24 | Summarize the main findings including the strength of evidence for each main outcome. | Discussion, paragraph2 |
| Limitations | 25 | Discuss limitations of study and outcome level. | Discussion, paragraph4 |
| Conclusions | 26 | Provide the implications for future research. | Discussion, paragraph4 |
| **FUNDING** | | |  |
| Funding | 27 | Describe sources of funding for the systematic review. | Funding |

*From:*  Moher D, Liberati A, Tetzlaff J, Altman DG, The PRISMA Group (2009). Preferred Reporting Items for Systematic Reviews and Meta-Analyses: The PRISMA Statement. PLoS Med 6(7): e1000097. doi:10.1371/journal.pmed1000097

For more information, visit: **www.prisma-statement.org**.

Page 2 of 2
