# Supplementary material for: Traditional Chinese Medicine as an adjunct therapy in the treatment of idiopathic membranous nephropathy: A systematic review and meta-analysis
Source: PLoS One. 2021 May 14;16(5):e0251131. doi: 10.1371/journal.pone.0251131 (PMC8121368; doi:10.1371/journal.pone.0251131)
Supplement: S1 File — (PDF) [file pone.0251131.s002.pdf]

PRISMA 2009 Flow Diagram

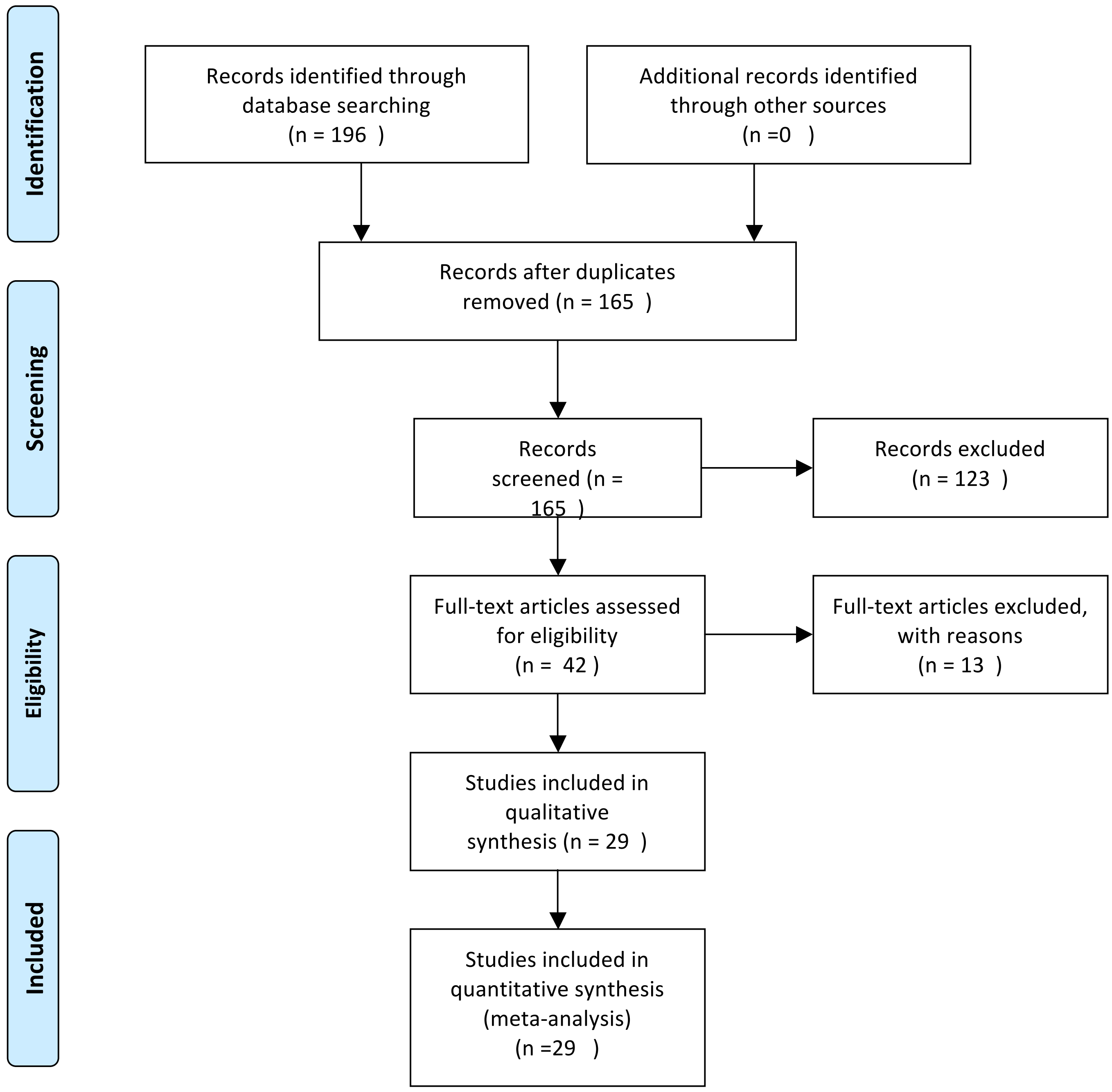

From: Moher D, Liberati A, Tetzlaff J, Altman DG, The PRISMA Group (2009). *P*referred *R*eporting *I*tems for *S*ystematic Reviews and *M*eta-*A*nalyses: The PRISMA Statement. PLoS Med 6(7): e1000097. doi:10.1371/journal.pmed1000097

For more information, visit www.prisma-statement.org.
